# A Screening Tool to Detect Chronic Critically Ill Cardiac Surgery Patients at Risk for Low Levels of Testosterone and Somatomedin C: A Prospective Observational Pilot Study

**DOI:** 10.7759/cureus.15298

**Published:** 2021-05-28

**Authors:** Ceressa T Ward, David W Boorman, Ava Afshar, Amit Prabhakar, Babar Fiza, Laura R Pyronneau, Amber Kimathi, Carmen Paul, Berthold Moser, Vanessa Moll

**Affiliations:** 1 Anesthesiology, Emory University School of Medicine, Atlanta, USA; 2 Pharmacy, Emory University Hospital Midtown, Atlanta, USA; 3 Pharmacy, Consumer Value Stores (CVS) Pharmacy, Atlanta, USA; 4 Food and Nutrition, Emory University Hospital Midtown, Atlanta, USA; 5 Rehab Therapy, Emory University Hospital Midtown, Atlanta, USA; 6 Anesthesiology and Critical Care, See-Spital Horgen, Horgen, CHE

**Keywords:** critical illness, somatomedin c, insulin-like growth factor 1, testosterone, growth hormone

## Abstract

Objective

The neuroendocrine response to critical illness is dichotomous as it is adaptive during the acute phase then transitions to maladaptive as critical illness becomes prolonged in 25-30% of patients. Presently, monitoring all critically ill patients for endocrinopathies is not the standard of care. However, given the negative impact on patient prognosis, a need to identify those at risk for endocrinopathies, may exist. Thus, a screening tool to identify endocrinopathies along the somatotroph and gonadal axes in a cardiothoracic surgery population was developed.

Methods

A prospective observational pilot study was conducted in two cardiothoracic surgery intensive care units (ICU) within a multi-site healthcare system. Total testosterone and somatomedin C levels were obtained from 20 adult patients who remained in the ICU for greater than seven days after cardiothoracic surgery and were tolerating nutrition, had a risk of malnutrition and a mobility score of moderate to dependent assistance.

Results

Twenty patients were included for descriptive analysis (seven females). Thirteen patients tested low for total testosterone, with males more likely to have a testosterone-related endocrinopathy as compared to females (100% vs. 0 to 43%, p = 0.0072). A higher proportion of low somatomedin C levels was found in females than males (57% vs. 31%); however, the difference was not statistically significant (p = 0.251).

Conclusions

The screening tool used in this pilot study accurately predicted low total testosterone in all men and reasonably predicted low somatomedin C in a majority of women. However, the ability of the tool to predict low total testosterone in women and low somatomedin C in men is less certain. A gender-specific screening tool might be necessary to predict hormonal deficiencies.

## Introduction

Significant research and subsequent advances in the management of critically ill patients has expedited the time to recovery and discharge from the intensive care unit (ICU) for a vast number of patients. However, 25-30% of ICU patients transition to a chronic phase of critical illness in which the stress-mediated neuroendocrine response becomes maladaptive [1-4]. Despite the provision of adequate nutrition support, ongoing hypermetabolism ensues with a minimum 10% loss of total body protein [1-3,5,6]. In addition to loss of lean muscle mass, chronic critical illness is defined as prolonged mechanical ventilation, development of myopathy and/or polyneuropathy, increased infectious complications, poor wound healing and the presence of endocrinopathy [1,2,6-9].

The neuroendocrine response to critical illness via the hypothalamic-pituitary axis (HPA) is biphasic (acute and chronic) and involves dysregulation of both the somatotroph and gonadal axes [1,2,4,10]. Growth hormone (GH), or somatotropin, is a large polypeptide that is essential for both direct and indirect metabolic functions throughout adulthood. In addition to promoting childhood physiologic growth, GH levels are also elevated in response to deep sedation, prolonged inadequate nutrition and stress (i.e., major trauma or surgery) [3,6,9,11]. After the initial onset of acute critical illness, there is a surge in hypothalamic-mediated pulsatile releases of GH, increased peripheral resistance to GH and reduced expression of GH receptors. This results in hyperglycemia secondary to insulin resistance and hepatic gluconeogenesis, enhanced immune response and increased lipolysis [1,2,4-6,10,12]. In addition to fatty acids being utilized as an energy source, increased proteolysis from skeletal muscle supplies 30% of calories needed to support the resting energy expenditure which can increase by as much as 40-60% [1,3,5,6,13,14]. The anabolic effects of GH such as amino acid uptake and protein synthesis are indirectly achieved via insulin-like growth factor (IGF-1) or somatomedin C, a product of hepatic synthesis [3, 5, 9]. In a hierarchical approach (survival versus death), the direct effects of GH take precedence while the anabolic effects of somatomedin C are suppressed [1,2,4-6,10].

The HPA also regulates the concentration of the gonadal steroid, luteinizing hormone (LH), which produces androgens (androstenedione and testosterone) via stimulation of both the ovaries and the adrenal gland in females as well as the Leydig cells in the testes of males. Physiologic levels of testosterone are necessary to increase amino acid utilization for protein synthesis in skeletal muscles and subsequent anabolism. In response to acute critical illness, decreased levels of testosterone have been noted secondary to cytokine-mediated inhibition of pulsatile LH release, desensitization of Leydig cells to LH and suppression of androgen production [1,3,4,15,16].

As critical illness transitions into a chronic phase, the predominant effects of GH partially subside while somatomedin C and testosterone levels continue to decline [1,2,4]. Concomitantly, these maladaptive processes contribute to an extensive depletion of net protein, a negative nitrogen balance and a loss of lean body mass. Evidence suggests a reduction of even 10% can provoke a negative sequelae inclusive of immune dysfunction, wound development and impaired healing, physical weakness (i.e., weakened diaphragmatic function) and/or death [1,3,5,6,9,14,15]. Almoosa et al. noted a correlation between the prevalence of hypotestosteronemia and the need for mechanical ventilation secondary to acute respiratory failure in 30 critically ill men [17]. This constellation of clinical features prolongs ICU length of stay (LOS) and gravely impacts patient prognosis with an ICU mortality rate of 15-20% [1,2,6]. In some chronically critically ill patients, the continued loss of protein and protracted muscle wasting persist despite the provision of adequate calories via nutrition support (i.e., enteral or parenteral) [1-3,5, 6].

Monitoring all medical-surgical ICU patients for endocrinopathies is not the standard of care at this time. However, for patients that transition into a phase of chronic critical illness, the need to identify those at risk for developing endocrinopathies, may exist. To assist in identifying post-surgical patients most likely at risk, an endocrinopathy screening tool was developed. Since cardiac surgery is defined as a risk factor for endocrinopathy and there is previous experience with correcting endocrinopathies in this population [18,19], the authors elected to pilot this tool in a cardiothoracic surgery (CTS) ICU. Thus, the objective of this study is to determine the ability of a newly designed screening tool to correctly identify endocrinopathies along the somatotroph and gonadal axes in chronically critically ill patients after CTS.

## Materials and methods

Study design

This prospective observational pilot study was conducted in two academic tertiary care CTS ICUs within a multi-site healthcare system from January 1, 2017 to October 31, 2017. This minimal risk study was approved by the Emory University Institutional Review Board (IRB#00092397). The Department of Pharmacy provided discretionary funds to pay for the laboratory tests needed for this study.

Patients

Utilizing the endocrinopathy screening tool, a manual review of the electronic medical record was conducted on any patient > 18 years old admitted to either of the two CTS ICUs for > 7 days. All patients meeting the following criteria were eligible for enrollment: status-post cardiac surgery requiring sternotomy, tolerating >75% of calculated resting energy expenditure as provided via oral, enteral and/or parenteral nutrition for at least 24 hours, at risk of malnutrition (defined as nothing by mouth and/or receiving <50% of required calories for ≥5 days) [20], and had a mobility score of moderate, maximum, or dependent assistance as defined by the Functional Independence Measure (FIM)^TM^ instrument [21]. For Medicare eligible patients, a G-code modifier indicating mobility that is 60%-100% impaired, limited, or restricted was also necessary for inclusion [22]. Written informed consent was obtained from either the patient or their legally authorized representative prior to enrollment. Informed consent was accomplished either in person or via phone. Consents obtained via the phone were witnessed by a licensed medical professional.

**Figure 1:**
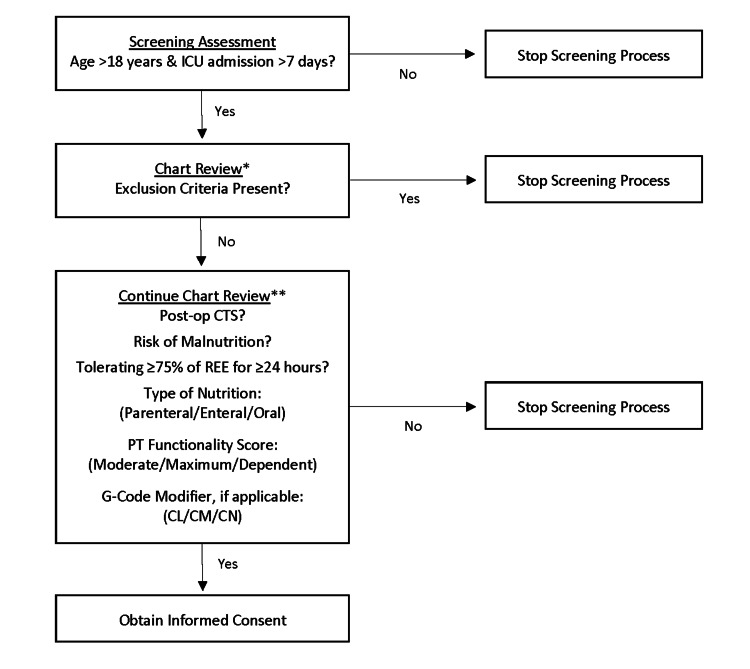
Somatotroph and Gonadal Axes Specific Endocrinopathy Screening Tool *Exclusion criteria: sepsis or septic shock; type 1 diabetes mellitus; chronic kidney disease stage G5 for greater than three months or end stage renal disease; chronic liver disease; history of organ transplant; active treatment for cancer; concurrent use of a glucocorticoid, sex hormone therapy (e.g., clomiphene, estradiol, testosterone) or dehydroepiandrosterone at any dose; or pregnancy. **Risk of malnutrition: nothing by mouth and/or inadequate nutrition (i.e., <50% of required calories for at least five days) [20].

Patients meeting the following criteria were excluded: sepsis or septic shock (defined as evidence of new or worsening infection plus two or more of the following criteria: temperature ≥38^◦^C or ≤35^◦^C; heart rate ≥110 beats per minute; respiratory rate ≥22 breaths per minute; and/or white blood cell count ≥12,000 cells/mm^3^ or ≤4 or >10% bands) [23]; type 1 diabetes mellitus; chronic kidney disease stage G5 [Glomerular filtration rate (GFR) < 15 mL/min/1.73 m^2^] for greater than three months or end stage renal disease [24]; chronic liver disease (elevated transaminases for at least six months, presence of cirrhosis, and elevated international normalized ratio [INR]); history of organ transplant; active treatment for cancer; concurrent use of glucocorticoid, sex hormone therapy (clomiphene, estradiol, testosterone), or dehydroepiandrosterone at any dose; or pregnancy (Figure 1).

**Figure 2:**
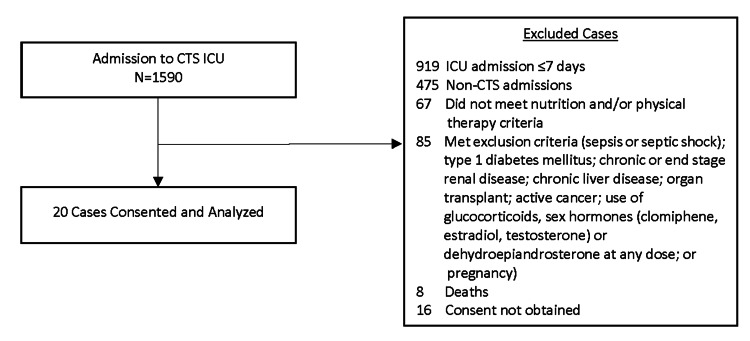
Exclusion Flowsheet

From January 1, 2017 to October 31, 2017, there were 1590 admissions in the CTS ICUs. Of those 1590 patients, 919 were excluded for ICU LOS that was at least seven days, 475 for non-CTS admissions, 67 who did not meet nutrition and/or physical therapy criteria, 85 who met exclusion criteria as previously stated, 16 in whom consent could not be obtained and eight deaths. In the end, 20 patients were consented and enrolled in this pilot study (Figure 2).

Data and laboratory collection

General demographics such as age, gender, race, height and weight were obtained from manual chart review. Blood was sampled within 24 hours of enrollment to collect total testosterone and somatomedin C levels. Patients were not required to be fasting at the time samplers were obtained. Blood draws were performed by the respective bedside nurse. The samples were obtained between the hours of 12:55AM and 9:55PM.

Total testosterone levels were quantified with chemiluminescent technology using the Abbott ARCHITECT i2000SR (Abbott, Abbott Park, IL, USA) immunoassay analyzer. Due to limitations in the ARCHITECT i2000SR test menu, samples for somatomedin C were shipped to ARUP Laboratories (Salt Lake City, UT, USA) and were processed using the Liaison® XL, a fully automated chemiluminescence analyzer manufactured by DiaSorin (Saluggia, Italy). The turnaround times were one to three business days for total testosterone (analyzed at this facility) and four to five business days for somatomedin C (analyzed at an outside facility).

Definition of endocrinopathy

Endocrinopathy was defined as having a subtherapeutic total testosterone level based on gender-specific parameters for adults and/or subtherapeutic somatomedin C levels as determined by age and gender-specific parameters (Table 1) [25].

**Table 1:** Therapeutic Somatomedin C and Total Testosterone Levels

	Males:	Females:
Somatomedin C	19-20 years: 281-510 ng/mL	19-20 years: 217-475 ng/mL
	21-30 years: 155-432 ng/mL	21-30 years: 87-368 ng/mL
	31-40 years: 132-333 ng/mL	31-40 years: 106-368 ng/mL
	41-50 years: 121-237 ng/mL	41-50 years: 118-298 ng/mL
	51-60 years: 68-245 ng/mL	51-60 years: 53-287 ng/mL
	61-70 years: 60-220 ng/mL	61-70 years: 75-263 ng/mL
	71-80 years: 36-215 ng/mL	71-80 years: 54-205 ng/mL
Testosterone, Total	Adult Males:	Adult Females:
	270-1100 ng/dL	10-80 ng/dL

Outcomes

The primary outcome of the study was the correct identification of endocrinopathy using the predefined endocrinopathy screening parameters. Patients with proven endocrinopathies were not supplemented as part of this study protocol. When an endocrinopathy was identified, the attending intensivist or their designated representative were notified and provided with recommendations for supplementing deficient levels.

Statistical analysis

Data were analyzed with SAS 9.4 (SAS Institute Inc., Cary, NC, USA). Patient hormone levels were marked as “normal,” “low” or “indeterminate”. Analysis of age and body mass index (BMI) versus low hormone levels was conducted with the Mann-Whitney U test. Analysis by gender was conducted with the Fisher’s Exact Test. Sensitivity power analysis was conducted with G*Power 3.9.1.4 (Düsseldorf, Germany) [26]. For purposes of continuous data analysis, any testosterone value below the detection level of 20 ng/dL was marked as 10 ng/dL. Three females had levels below the detection threshold of 20 ng/dL. Therefore, whether they were within normal range (10-80 ng/dL) could not be assessed, and they were removed from the categorical data analysis except as noted below.

## Results

Seven out of 20 patients were female and seven were Black. The median age was 67 years (Interquartile range (IQR) 54.5-72.5), median BMI 29.7 (IQR 27.1-33.5) (Table 2).

**Table 2:** Demographics of Study Group IQR: Interquartile range; BMI: Body mass index; Af. Amer.: African American

Characteristic	Patients (N = 20)
Age, median (IQR)	67 (54.5-72.5)
BMI, median (IQR)	29.7 (27.1-33.5)
Sex, female, n (%)	7 (35%)
Race, Black, n (%)	7 (35%)

Total testosterone

Neither age nor BMI was significantly associated with low total testosterone. The proportion of subjects whose total testosterone and somatomedin C levels were “Low” or “Normal,” adjusted for age and gender is shown in Table 3.

**Table 3:** Proportion of Study Group with “Low” Hormone Levels, Total and by Sex Proportion of subjects whose testosterone and Somatomedin C levels were “Low” or “Normal,” adjusted for age and gender, as defined above. Of note, when separating out the data by sex, the screening process appears to detect low testosterone levels in men very well, but less well in females. Alternatively, the screening process appears to detect low somatomedin C in females reasonably well, but less well in males. LCL: Lower control limit; UCL: Upper control limit.

Hormone	Low	Normal	Indeterminate	Total	Proportion Low	95% LCL	95% UCL
Testosterone	13	4	3	17	0.765	0.501	0.932
Testosterone, Female	0	4	3	4	0.00	0.000	0.602
Testosterone, Male	13	0	0	13	1.00	0.753	1.00
Somatomedin C	8	12	0	20	0.40	0.185	0.615
Somatomedin C, Female	4	3	0	7	0.571	0.184	0.901
Somatomedin C, Male	4	9	0	13	0.3077	0.0909	0.6143
Either, Female & Male	17	2	1	20	0.895	0.669	0.987

Males were more likely to have total testosterone levels below the normal range (13/13 males vs. 0/5 females, p < 0.0004, Fisher’s Exact Test) (Table 4).

**Table 4:** Hormone Levels by Sex ^a^ Fisher Exact Test ^b^ Three women indeterminate at < 20. Top row: three women omitted. Bottom row: women counted as Low testosterone. Both are statistically significant.

Hormone	Laboratory Value	Sex		p-value ^a^
		Female	Male	
Testosterone	Low, n (%)	0 (0%) or 3 (43%)^b^	13 (100%)	0.0004 or 0.0072^b^
	Normal, n (%)	4 (80%) or 4 (57%)	0 (0%)	
Somatomedin C	Low, n (%)	4 (57%)	4 (31%)	0.251
	Normal, n (%)	3 (43%)	9 (69%)	

Three females had testosterone levels below the detection threshold of 20 ng/dL, but it could not be determined if they were below normal range of 10-80 ng/dL, and thus were removed. If all three females had low total testosterone, then 43% would have low total testosterone, which is still significant at p = 0.0072 (Table 4). Because the sample size was low, conclusions cannot be drawn about total testosterone levels among females.

Somatomedin C

**Figure 3:**
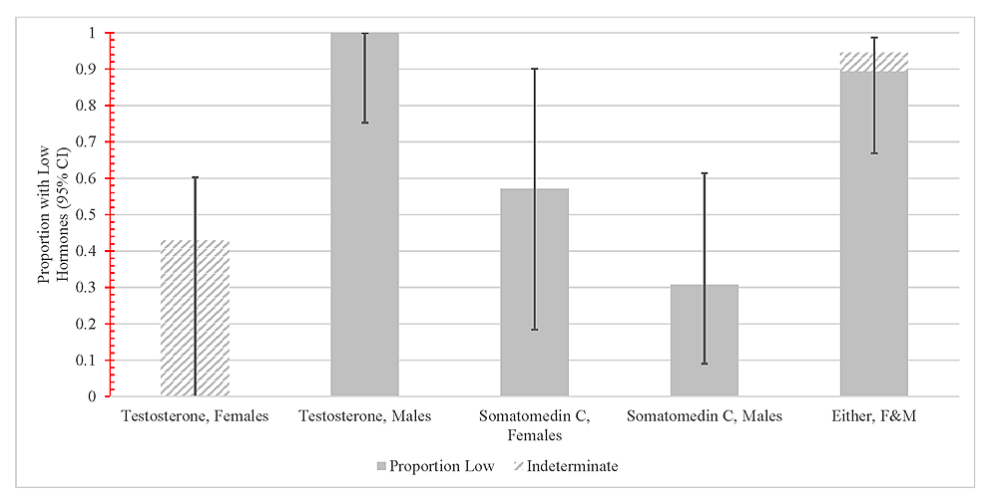
Proportion of Screened Female and Male Subjects who were Accurately Assessed as Having Low Hormone Levels The screening process appears to perform excellently for males to detect low testosterone, but less well for females. Alternatively, the screening process appears similar for Somatomedin C by sex. Overall, 89.5% of this male-heavy samples were correctly identified with low hormones in either testosterone, somatomedin C or both. F: Female; M: Male

**Figure 4:**
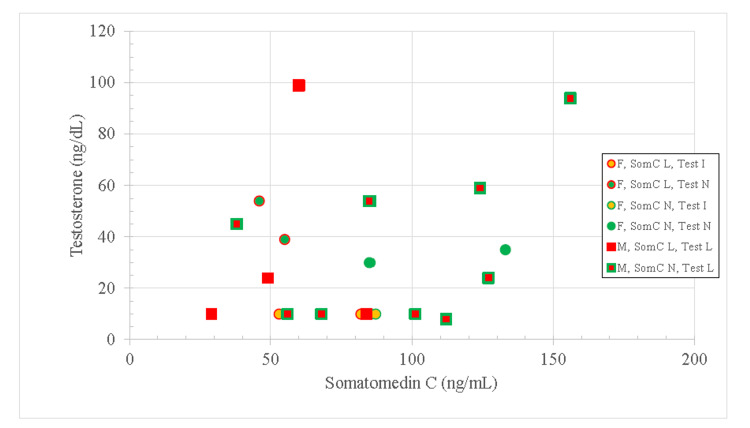
Distribution of Hormone Levels by Gender Hormone levels of subjects by gender, female (circles), and male (squares). Hormone levels are marked as low (red), normal (green), or indeterminate (yellow). Testosterone levels are marked as the interior color and somatomedin C as the exterior. Three females had testosterone levels below the detection threshold of 20. Therefore, whether they were within normal range (10-80 ng/dL) could not be assessed, they were marked as “Indeterminate” and the level was set at 10 ng/dl. Results show that only two of the twenty had normal levels in both hormones, with one normal in somatomedin C, but indeterminate in testosterone. F: Female; M: Male; L: Low; I: Indeterminate; N: Normal; SomC: Somatomedin C; Test: Testosterone.

Low somatomedin C was not associated with age or BMI. Four out of seven female patients (57%) and four out of 13 male patients (31%) had low levels of somatomedin C but the difference was not statistically significant (p = 0.251). Table 3 and Figure 3 show the distribution of somatomedin C and total testosterone by gender. Somatomedin C and total testosterone levels by gender have also been provided (Figure 4).

## Discussion

The screening tool identified 13 out of 17 patients with low total testosterone levels. When separating out the data by gender, the screening process exposed low total testosterone levels in males (13/13) very well, but less well in females (3/7 indeterminate). The screening tool seemed to detect low somatomedin C better in females (4/7) than males (4/13). Treatment of hormonal deficiencies in critically ill patients has not been reflected in guidelines. As such, the decision to supplement these deficiencies was left to the discretion of the attending intensivist. Four patients received varying durations of therapy for hormone deficiencies; three patients received monotherapy with oxandrolone and one patient received both somatotropin and oxandrolone. Of these four patients, two patients were discharged, with a tracheostomy, to an acute skilled nursing facility, one patient was discharged to home and one patient died.

During the acute phase of critical illness or after major procedures such as CTS, the endocrine response to physiologic stress in males is impaired as evidenced by a rapid decline in testosterone levels. As the critical illness persists, LH concentrations are reduced and testosterone levels become undetectable [4,16,18]. Given that serial levels were not obtained at both baseline and 24 hours post-op, acute declines in testosterone were not observed in this study. However, after seven days in the ICU, status-post surgery, testosterone deficiency was reported. While less than half of the females in the study group had undetectable levels, all males had levels well below the lower threshold of normal (270 ng/dL). Limited data suggest that concentrations of LH in postmenopausal women are also reduced in response to critical illness as well [4]. Since the total testosterone levels could not be quantified below 20 ng/dL, conclusive statements regarding the impact of critical illness status-post CTS on total testosterone levels in females cannot be derived at this time.

For patients with a prolonged course of post-surgical immobility and protein malnutrition, the presence of testosterone deficiency can manifest as hypercatabolism, decreased lean muscle mass and reduced physical functionality. In males with low free-testosterone levels, it has been suggested that functionality likely declines by 57% [4,18]. To the authors’ knowledge, the degree of physical decline in females secondary to critical illness has not been established. Though the lower endocrinopathy levels in our study were not all statistically significant, it is worth noting that all patients had impaired functional mobility scores ranging from moderate assistance to completely dependent.

Progressive declines in somatomedin C in response to prolonged critical illness has been well defined. Similarly, the findings of this study demonstrate the presence of hyposomatotropism in post-surgical patients with a prolonged ICU LOS and evidence of debilitation despite the provision of nutrition support therapy. In a case series of four patients in a surgical ICU, subtherapeutic somatomedin C serum concentrations were noted after greater than 19 days in the ICU with concomitant debilitation and/or dependency on mechanical ventilation [6]. A small study of 23 cardiac surgical patients found growth hormone and/or testosterone levels to be deficient when obtained after >7 days in the ICU [19]. It has been further noted that hyposomatotropism is more apparent in males as opposed to females likely because females experience higher stress-induced surges of GH levels [1,6,11]. However, a correlation between this finding and outcomes has yet to be confirmed. In this study, there was no correlation between the number of postoperative ICU days and patients with subtherapeutic somatomedin C and/or total testosterone levels. Deficient hormone levels were noted as early as postoperative day 8 and as late as postoperative day 83.

Herein, this study suggests that the use of a screening tool may be useful in identifying patients with some degree of somatotroph and/or gonadal axes specific endocrinopathies. With a wide range of ICU days by which endocrinopathies were identified, lab monitoring in patients with an ICU LOS greater than seven days and debilitation despite the provision of enteral and/or parenteral nutrition may be reasonable. Based on the findings of this study and the available literature, the appropriateness of obtaining both a somatomedin C and total testosterone level to define endocrinopathies in all at risk patients is questionable.

Hypotestosteronemia is also suggested to be the result of advancing age, physiological stress due to major surgery (e.g., cardiac surgery), and the presence of cardiovascular disease. Data further suggest that elderly males with a history of depressed ejection fraction (<40%) placed on cardiopulmonary bypass for cardiac revascularization are at risk of subsequent declines in the production of both testosterone and somatomedin C [18,27]. In a prospective study of 54 patients, 35 of whom were males, significant declines in testosterone levels were noted within 24 hours of injury (e.g., traumatic brain, general surgery, myocardial infarction). The authors theorized that hypotestosteronemia was potentially due to accelerated hormone metabolism, reduced production and inadequate or refractory gonadal response to stimulation [28]. Similarly, Spratt further speculates that an increase in testosterone clearance may exist; however, a larger study population is needed to confirm these findings [15]. Given the multiple confounding factors impacting testosterone levels (i.e., age, male gender, history of cardiac disease, and timing of specimen collection) and the limited data in women, use of total testosterone levels may potentially lead to a false positive diagnosis of endocrinopathy if obtained after physiological stress. These authors would only recommend the use of testosterone levels if baseline levels were obtained prior to physiological stress along with a follow-up total testosterone level both 24 hours post-operative and when critical illness is prolonged. To better assess for potential impacts of critical illness on testosterone levels, availability of age-specific testosterone levels may prove to be more useful. In this study, the authors only had access to testosterone levels which provide a broad range for normal in male patients at least 18 years old.

The role of hormone supplementation, specifically recombinant human GH (rhGH) and anabolic steroid therapy, in critical illness remains uncertain. However the literature presented thus far has utilized a broad scope of non-standard criteria to identify which patients should receive treatment for endocrinopathies. Thus, the challenge remains to identify the specific patients likely to benefit from supplementation and therefore, a standardized approach to identifying patients most likely at risk for developing endocrinopathies is warranted. The development of this endocrinopathy screening tool is a novel approach and attempt at harmonizing potential risk factors into an algorithmic method to identify patients at risk for somatotropic and/or gonadotropic specific endocrinopathies. Further studies to formulate and validate such a tool is a warranted first step towards ensuring that the appropriate patients receive supplementation.

Limitations of the study

Due to limited funding available to pay for laboratory tests, the small sample size included in this pilot study represents a major limitation. Hormone data was not collected from patients who did not pass the screening process, which means the sensitivity and specificity of the screen could not be calculated. Data analysis of this pilot study was delayed due to logistical issues impeding a larger trial to validate the screening tool. However, the findings of this pilot study within one healthcare system may justify the provision of additional funding for a larger trial. Although cardiac surgery has been identified as a risk factor for testosterone deficiency, it is also worth noting that the CTS ICU is a specialized area with high turnover and the number of patients qualifying for endocrinopathy testing is limited. Therefore, future studies in a broader ICU population (e.g., medical/surgical) may substantiate these findings.

The inconsistency in time of blood draws for total testosterone represents a limitation in the accuracy of raw testosterone values. Diurnal variations in testosterone can be 30-35% in young men, dropping to 10% in men at age 70, with highest levels seen in the early morning compared to lowest in late evening; it does not seem to vary significantly in women [29-31]. With a maximum value of 99 ng/dL in our sample compared to men’s threshold of 270 for “normal” levels, there is essentially no chance of this affecting the analysis or conclusions of this study. Standardizing the time and serial lab draws to reduce the impact of errors in specimen handling or lab errors could be important for a larger, future study. As this study was purely designed to identify patients with somatotrophic and/or gonadotrophic specific endocrinopathies, clinical outcomes secondary to hormone supplementation were not documented.

Treatment of chronic critically ill patients

The current standard of care for the chronically, critically ill population remains as follows: addressing any acute issues, providing optimal nutrition with sufficient proteins, and consulting both physical and occupational therapy to promote strengthening and mobility. With an expanding wealth of knowledge and understanding about neuroendocrine alterations during critical illness, the use of hormone supplementation in patients with chronic critical illness would appear to be the obvious solution in properly identified patients. Therapies such as parenteral rhGH for somatomedin C deficiency and anabolic steroids such as parenteral testosterone cypionate or oral oxandrolone for testosterone deficiency have been trialed. Unfortunately, due to a number of studies with conflicting findings, the use of exogenous hormone supplementation remains controversial.

Two prospective, multicenter, randomized, placebo-controlled trials identified a higher mortality rate, compared to placebo, when high doses of rhGH were administered for a maximum 21 days to 532 medical-surgical ICU patients (p < 0.001 for both studies) [32]. The outcomes of these studies have been attributed to the use of rhGH doses 10-20 fold higher than recommended; use of rhGH in acutely ill patients in whom the initial inflammatory response had not fully resolved (i.e., timing of rhGH initiation); and the preponderance for hyperglycemia in the rhGH group [1,6,32]. Since the publication of studies by Takala et al., the potential benefits of rhGH therapy in carefully selected patient populations have continued to be evaluated. A prospective, double-blind, placebo-controlled trial study by Zhang et al., randomized 48 patients to receive either 0.15 IU/kg of rhGH or placebo from postoperative day 3 to 10 after elective abdominal surgery. The investigators concluded that patients in the treatment group had a significantly higher nitrogen balance compared to placebo (p < 0.05). Although hyperglycemia was higher in the rhGH group (p < 0.05), there were no reports of sepsis or death [33]. Four patients in a surgical ICU with subtherapeutic levels of IGF-1 were treated with two courses of rhGH after the onset of chronic critical illness. After completing both courses of rhGH, the patients were liberated from mechanical ventilation, tolerating an oral diet, and were either discharged to a long-term acute-care facility or a medical floor. Additionally, three patients were also noted to have some degree of physical functionality (i.e., sit to stand, walk) [6]. Fifty-three patients in a surgical ICU with prolonged mechanical ventilation, defined as greater than 20 days, received an average 37 days of rhGH (doses ranging: 0.04-0.14 mg/kg/day) for diaphragmatic weakness and pulmonary disease. This study reported that 81% of patients were liberated from the ventilator [34]. Conversely, in a placebo-controlled study of 20 patients requiring mechanical ventilation for more than seven days, a 12-day course of rhGH (dosed at 0.14 mg/kg/day) did not hasten the time to ventilator weaning [35]. The use of rhGH, oxandrolone, and/or testosterone therapy in 23 chronic, critically ill post-cardiac surgical patients with known hormonal deficiencies resulted in a significant reduction of in-hospital mortality when compared to a similar surgical unit within the same healthcare system [19].

The most beneficial strategy to help patients overcome chronic critical illness might be a multi-modal approach consisting of hormone replacement, optimized nutrition, and structured exercise (physical therapy) [36]. One anabolic treatment option is oxandrolone, which is an FDA-approved testosterone analogue for treating muscle weakness in ICU patients. Combined with structured exercise, this approach has shown clinical benefit [37,38]. A case series describes the potential role and benefit of nandrolone (an injectable anabolic steroid) for weight gain and ICU-related myopathy for patients in the recovery phase of critical illness [39]. While the combination of an anabolic agent with early exercise and adequate nutrition in the ICU is likely the key to optimize muscle strength and functional outcomes for recovering critically ill patients [36], no large randomized controlled trials have yet led to guidelines addressing anabolic agent supplementation. Similarly, no guidelines address the use of growth hormone supplementation in recovering critically ill patients. Randomized controlled trials are needed to address this gap. The goal of this pilot project was to identify patients at risk for a somatotroph and/or gonadal axes specific endocrinopathies. Without treatment guidelines, we left the decision to supplement hormonal deficiencies to the responsible intensivist.

## Conclusions

The screening tool was most advantageous in identifying a testosterone endocrinopathy in recovering critically ill male patients. The deficiency in somatomedin C levels appears better identified in females. The use of this screening tool poses the first step to recognize patients at risk. Larger studies will have to further evaluate gender differences and optimization of a gender-based screening strategy. Supplementation therapy with rhGH and anabolic steroids in conjunction with structured exercise and optimized nutrition seems to lead to a clinical benefit in functional outcomes. Randomized controlled trials identifying and treating these deficiencies are necessary to eventually develop guidelines. Development and implementation of a standardized protocol to detect somatotrophic and/or gonadotropic specific endocrinopathies is a first step in improving the care of patients with chronic critical illness.
